# Accuracy of speckle tracking in the context of stress echocardiography in short axis view: An in vitro validation study

**DOI:** 10.1371/journal.pone.0193805

**Published:** 2018-03-27

**Authors:** Amir Hodzic, Boris Chayer, Diya Wang, Jonathan Porée, Guy Cloutier, Paul Milliez, Hervé Normand, Damien Garcia, Eric Saloux, Francois Tournoux

**Affiliations:** 1 Research Unit of Biomechanics and Imaging in Cardiology, University of Montreal Hospital Research Center, Montréal, Québec, Canada; 2 Department of Cardiology, Echocardiography Laboratory, University Hospital Center, Caen, France; 3 Inserm Comete, Unicaen, University of Caen Normandy, Caen, France; 4 Laboratory of Biorheology and Medical Ultrasonics, University of Montreal Hospital Research Center, Montréal, Québec, Canada; 5 Department of Cardiology, Echocardiography Laboratory, Hospital of the University of Montréal, Montréal, Québec, Canada; University of Bern, University Hospital Bern, SWITZERLAND

## Abstract

**Aim:**

This study aimed to test the accuracy of a speckle tracking algorithm to assess myocardial deformation in a large range of heart rates and strain magnitudes compared to sonomicrometry.

**Methods and results:**

Using a tissue-mimicking phantom with cyclic radial deformation, radial strain derived from speckle tracking (RS-SpT) of the upper segment was assessed in short axis view by conventional echocardiography (Vivid q, GE) and post-processed with clinical software (EchoPAC, GE). RS-SpT was compared with radial strain measured simultaneously by sonomicrometers (RS-SN). Radial strain was assessed with increasing deformation rates (60 to 160 beats/min) and increasing pulsed volumes (50 to 100 ml/beat) to simulate physiological changes occurring during stress echocardiography. There was a significant correlation (R^2^ = 0.978, P <0.001) and a close agreement (bias ± 2SD, 0.39 ± 1.5%) between RS-SpT and RS-SN. For low strain values (<15%), speckle tracking showed a small but significant overestimation of radial strain compared to sonomicrometers. Two-way analysis of variance did not show any significant effect of the deformation rate. For RS-SpT, the feasibility was excellent and the intra- and inter-observer variability were low (the intraclass correlation coefficients were 0.96 and 0.97, respectively).

**Conclusions:**

Speckle tracking demonstrated a good correlation with sonomicrometry for the assessment of radial strain independently of the heart rate and strain magnitude in a physiological range of values. Though speckle tracking seems to be a reliable and reproducible technique to assess myocardial deformation variations during stress echocardiography, further studies are mandated to analyze the impact of angulated and artefactual out-of-plane motions and inter-vendor variability.

## Introduction

Stress echocardiography is commonly used for ischemic or valvular diseases assessment [1–3]. Global and regional cardiac functions are key parameters reported during this test. Their assessment is mostly based on visual interpretation of the B-mode cineloop with a high risk of intra- and inter-observer variability, depending on the reader’s experience [4]. Twenty years ago, tissue Doppler imaging-derived strain (TDI-strain) was proposed as a technique to provide more objective global and regional assessment of the myocardial function at rest and during stress, with acceptable spatial and good temporal resolution [5,6]. Because of its angle dependency (since it is a Doppler-based technology) limiting which myocardial segments might be studied and a poor signal-to-noise ratio [7,8], TDI-strain has not significantly been adopted in routine clinical practice in stress echocardiography. In fact, the accuracy and reproducibility of this tool are felt by clinicians to be inferior to the eye of an experienced echocardiographer.

Ten years ago, a speckle tracking tool based on gray-scale image processing emerged as an exciting alternative for assessing global and regional myocardial deformations. Speckle tracking analysis is based on a block-matching approach of the speckle patterns within the myocardium. It allows strain assessment of myocardial segments relatively independent of their position with respect to the ultrasound beam direction [9,10]. The accuracy of speckle tracking-strain in either experimental models [11,12] or patients [13–15] has already been demonstrated, mostly during studies with a constant and slow heart rate. To be accurate, the algorithm requires a relatively low frame rate (40–80 Hz) to identify and track a specific pattern. While the frame rate/heart rate ratio at rest is adequate (almost 70 frames per cardiac cycle for example if the heart rate is 70 beats per minute and the frame rate is 80 Hz), the algorithm’s accuracy is not guaranteed for much higher heart rates such as those observed during stress tests (only 35 frames per cardiac cycle at 140 beats per minute for a frame rate of 80 Hz). The undersampling at high heart rates could lead to a lower accuracy of strain estimates because of speckle decorrelation due to the large movement between consecutive frames or aliasing if displacements become larger than the block-matching kernel size. Moreover, it is possible that the importance of such limitation might be different between a normal and a failing heart. Several studies have previously highlighted the additional value of the use of speckle tracking during a stress echocardiography [16–20]. However these in vivo studies cannot conclude that the observed changes in speckle tracking myocardial strain values during the adrenergic stimulation are accurate since those changes might be explained by either a technical artifact (because of the rapid heart rate) or the inotropic effect of the stimulation, or both.

In vitro studies are usually required to set the exact limits of an algorithm in a controlled environment by altering either individual or composite parameters of the studied imaging tool. Our study aimed to determine in a dynamic tissue-mimicking phantom the accuracy of speckle tracking-strain to assess tissue deformation for different heart rates and strain magnitudes, compared to sonomicrometry as a gold standard.

## Materials and methods

### In vitro model

**Fig 1:**
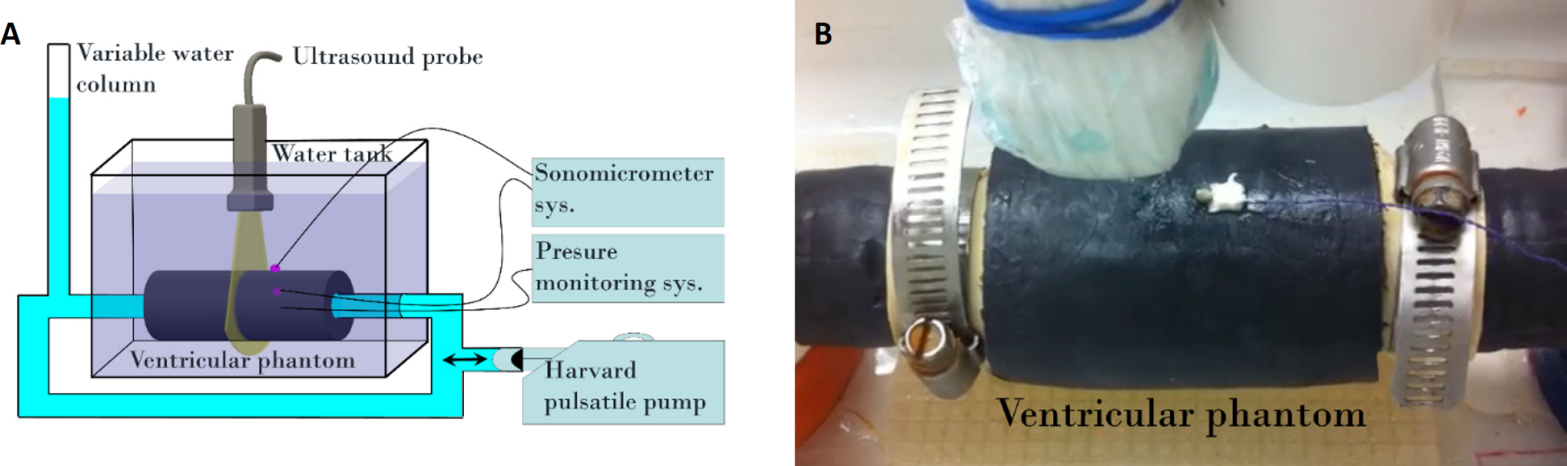
(A) Illustration of the experimental setup and (B) photo zoomed on the phantom model.

We used a dynamic tissue-mimicking phantom model previously used for strain validation [21]. Briefly, the phantom was prepared using 10% by volume of polyvinyl alcohol dissolved in pure water and mixed with 1% by weight of graphite particles to simulate the stiffness and acoustic backscatter properties of the myocardial tissue. The gel was molded into a homogeneous hollow cylinder approximately 15 cm in length, 5 cm in outer diameter and 3.5 cm in lumen diameter, with a wall thickness of 1.5 cm. Gel solidification and polymerization were induced by two freezing-thawing cycles in a temperature-controlled chamber [22]. The gel was connected at each end to an adjustable external pulsatile blood pump (Harvard Apparatus, Holliston, MA, USA) with polyvinyl chloride tubing. The pulsed volume of water inside the cylinder was set at 50, 70 and 100 ml/beat to generate different magnitudes of radial deformation and simulate a failing versus a normal heart. For each volume, the pulse rate of the pump was increased from 60 to 160 beats/minute in increments of 10 beats. A total of 10 following experiments were conducted: 4 using a pulsed volume of 50 ml/beat, 3 using 70 ml/beat and 3 using 100 ml/beat. For each pulsed volume, the pump rate was increased from 60 to 160 beats/minute. This resulted in a total of 110 measures of radial strain assessed by echocardiography and sonomicrometry. A pressure catheter introduced into the cylinder was used as a common temporal reference for the sonomicrometric and echocardiographic recordings. A water column was added to generate a mean pressure between 80 and 100 mmHg in the phantom to mimic the systemic blood pressure. An illustration and a photo of the experimental setup are shown in Fig 1. A video of the setup under dynamic conditions has been added under supporting information (S1 video).

### Strain measurements by sonomicrometry

Two sonomicrometer crystals of 2 mm in diameter (Sonometric Corporation, London, Ontario, Canada) were sutured radially to the internal and external surfaces of the upper wall so that mechanical deformations of the gel were not affected by the presence of crystals (Fig 1). Sonomicrometry was used as a gold standard for radial strain measurements (RS-SN). RS-SN(t) was computed by dividing the change in distance (d) between the two crystals by the distance between those crystals under static hydrodynamic pressure (*i*.*e*., with the pulsatile pump off; the radial strain was computed as (d(t)-d(0))/d(0)). For comparison of sonomicrometric data with echocardiographic data, half of the thickness of each crystal was subtracted from the inter-sonomicrometer distances. Sonomicrometric data were acquired immediately before echocardiographic measurements to avoid ultrasound interference due to potential overlapping frequency bands between these two systems.

### Strain measurements by speckle tracking echography

**Fig 2:**
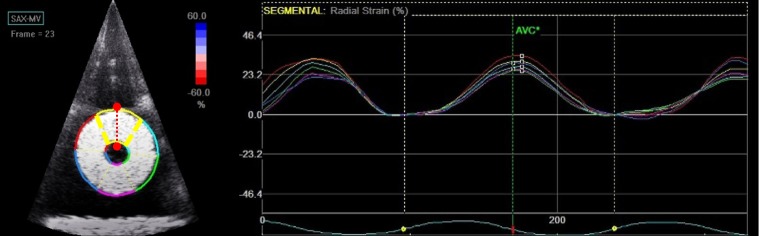
Speckle tracking echography of the phantom in short axis view. The software algorithm automatically segments the phantom into six regions. The positions of sonomicrometer crystals at the internal and external surfaces of the phantom are indicated in red. Peaks of strain versus time curves corresponding to the six segments are obtained (here for radial strain).

The phantom was immersed in water and imaged using a commercially available Vivid q machine equipped with a 2.5-MHz probe (M4S-RS) (General Electric Medical System, Milwaukee, WI, USA). The transducer was fixed perpendicularly to the long axis of the gel cylinder and approximately 2 cm lateral to the sonomicrometer positions to avoid imaging artifacts (Fig 1). Radial strain was analysed from a conventional two-dimensional echographic greyscale short axis view with a frame rate between 60 and 80 Hz (as recommended to have an acceptable spatial and temporal definitions of the myocardial tissue when using speckle tracking analysis) [23]. Standard clinical settings for gain and compression were used during image acquisition. In post-processing, we used a speckle-tracking algorithm incorporated into an EchoPAC workstation (BT12, GE Medical). Segmentation was performed semi-automatically at end diastole (end of radial expansion) and divided into six segments. The tracking quality was visually inspected and confirmed for each segment. The speckle tracking software was able to compute strain automatically from the frame-to-frame radially and transversally directed displacements of speckle patterns throughout the entire cycle. The radial deformation curve from the upper segment obtained by the speckle tracking analysis was used for comparison with the gold standard since the sonomicrometers were located within the same region. The maximum radial strain value was considered in this evaluative study and was obtained from the positive peak value of the radial strain curve (RS-SpT). Three consecutive cycles were averaged for comparison with sonomicrometric data. An example is shown in Fig 2 and S2 Video.

### Reproducibility

Intra and inter-observer variability in the measurement of speckle tracking-derived strain were assessed in 3 sets of data (n = 33 measures) randomly selected and including all pulsed rates between 60 and 160 beats per minute. Inter-observer variability was obtained by two independent observers. The intra-observer variability was tested using two sets of measurements performed at least 2 weeks from each other.

### Statistical analysis

Statistical analyses were performed using MedCalc for Windows, version 13 (MedCalc Software, Ostend, Belgium). The agreement between sonomicrometry and speckle tracking-strains was assessed by the Bland and Altman method [24] and the correlation coefficient. One-way and two-way analysis of variance tests were used to study the influence of the pump rate and strain magnitude on speckle tracking measurements. The intraclass correlation coefficient (ICC) was calculated as a measure of the intra- and inter-observer variability of the speckle tracking-strain method. A P value <0.05 was considered significant.

## Results

### Feasibility and reproducibility

The assessment of radial strain was feasible at each pump rate (from 60 to 160 beats/minute) and pulsed volume (from 50 to 100 ml/beat) that we tested. At low pulse rates (≤100 beats/minute, n = 15 measures), the ICC for speckle tracking-strain were 0.96 (95% CI, 0.9–0.99) and 0.97 (95% CI, 0.92–0.99) for the intra- and inter-observer variability, respectively. At higher pulse rates (>100 beats/minute, n = 18 measures) ICC were 0.95 (95% CI, 0.86–0.98) and 0.98 (95% CI, 0.95–0.99) for the intra- and inter-observer variability, respectively. Strain value ranges between the lowest rates (8.9%–30.1%) and fastest rates (10.4%–32.2%) were not statistically significant (P = 0.3).

### Validation of speckle tracking echography

**Fig 3:**
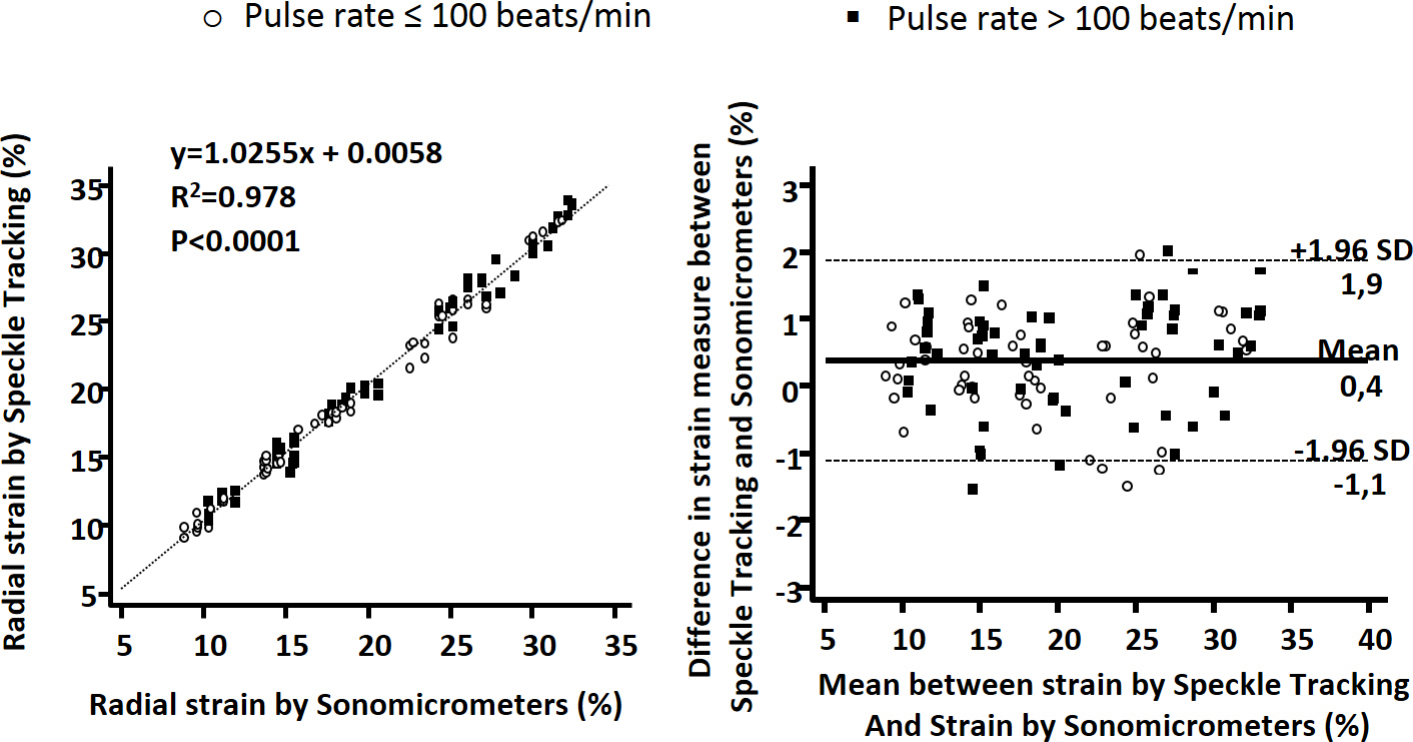
Correlation (left) and Bland-Altman analysis (right) of radial strain measurements between speckle tracking and sonomicrometers.

The correlation between echocardiographic and sonomicrometric pooled measurements was highly significant (R^2^ = 0.98, P <0.001) with a slope close to 1 (Fig 3). The Bland Altman analysis confirmed a close overall agreement (bias ± 2 SD, 0.39 ± 1.5%) (Fig 3).

A detailed comparison of strain measurements by the two methods at different loading and pump rate conditions is shown in Table 1.

**Table 1:** Comparison of the mean radial strain values by sonomicrometry and by speckle tracking.

Pump rate (beats/min)	50 ml/beat		70 ml/beat		100 ml/beat		P value
	Sono (%)	Echo (%)	Sono (%)	Echo (%)	Sono (%)	Echo (%)	
**60**	11.5 ± 2.6	11.8 ± 2.7	19.2 ± 4.7	20.1 ± 4.5	26 ± 3.7	26.8 ± 4	NS
**70**	11.8 ± 3	12.3 ± 2.3	20.1 ± 3.7	20.9 ± 4.7	26.6 ± 4.8	26.4 ± 5.2	NS
**80**	11.9 ± 2.2	12.4 ± 2.4	19.8 ± 4	20.2 ± 4.4	26.2 ± 3.6	26.2 ± 4.7	NS
**90**	12.1 ± 2	12.6 ± 2.5	20.6 ± 4	20.8 ± 5	27.1 ± 3.6	26.2 ± 4.7	NS
**100**	12.7 ± 2.2	12.8 ± 2.5	21.2 ± 4.3	21.4 ± 4.5	27.4 ± 4	27.2 ± 4.6	NS
**110**	12.7 ± 2.2	13.5 ± 2.7	21.2 ± 4.2	22.3 ± 5	27.7 ± 3.6	27.9 ± 4.3	NS
**120**	12.9 ± 2.2	13.4 ± 2.6	21.2 ± 4.3	21.9 ± 4.8	28.2 ± 3.9	28.9 ± 4.4	NS
**130**	13.2 ± 2.7	13.4 ± 2.3	21.8 ± 4.5	22.3 ± 4.9	28.9 ± 3.6	29.4 ± 3.7	NS
**140**	13.1 ± 2.3	13.7 ± 2.1	21.8 ± 4.5	22.4 ± 4.7	29.3 ± 3.7	30 ± 3.6	NS
**150**	13.1 ± 2.4	13.5 ± 1.7	21.8 ± 4.7	21.9 ± 5.4	28.9 ± 3.2	29 ± 3.9	NS
**160**	13.2 ± 2.5	13 ± 1.6	22.1 ± 5.2	22.5 ± 6.2	29.4 ± 3.8	29.7 ± 3.5	NS

Values are expressed as mean ± SD. Sonomicrometry and echo data were compared using unpaired t-test. NS = non-significant, P >0.05.

**Fig 4:**
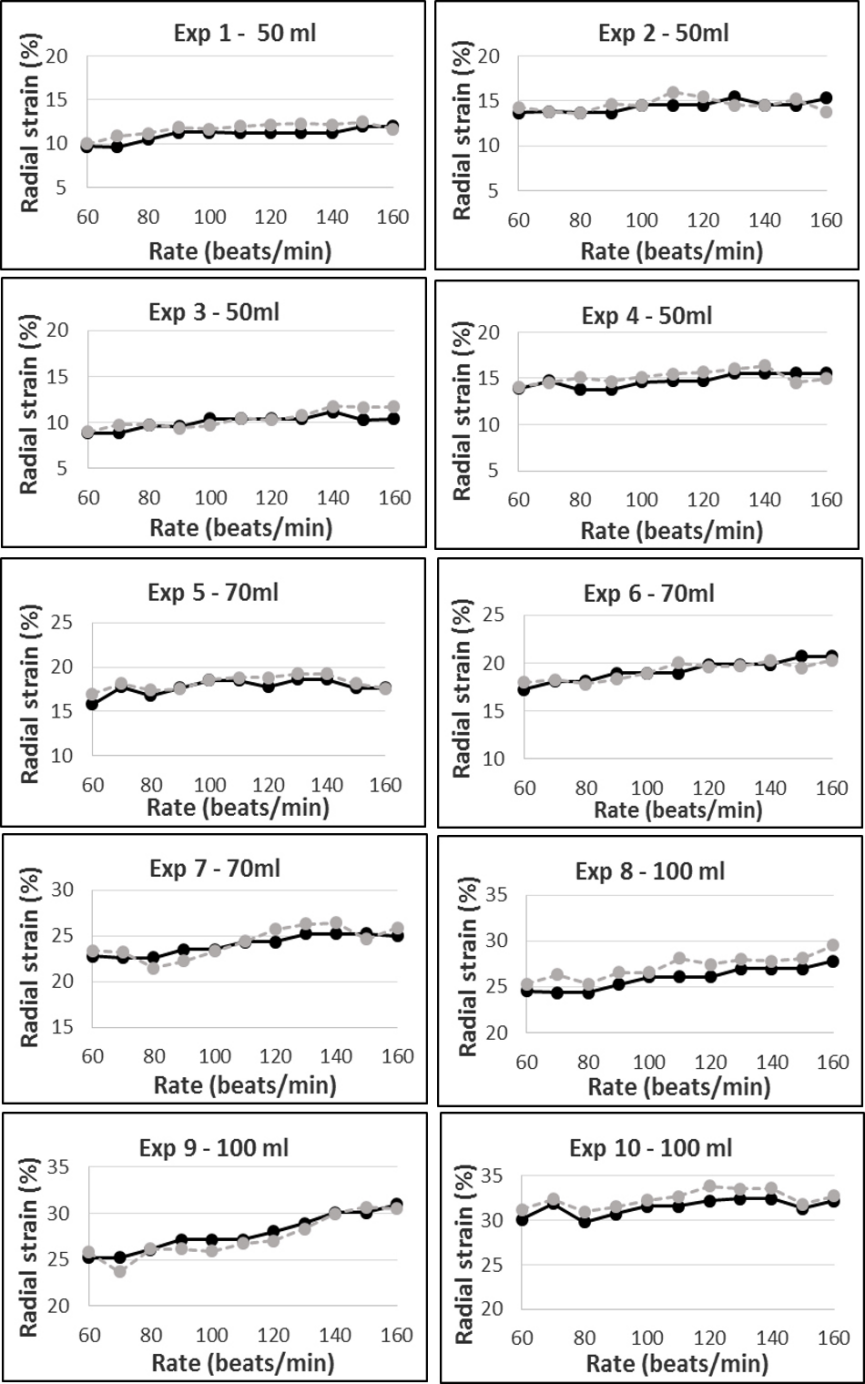
Graphic representation of the total experimental data sets (Exp1-10). These data were obtained from simultaneous measurements of the upper segment radial strain by sonomicrometers (black full lines) and by speckle tracking (grey dotted lines), recorded for each pump rate (from 60 to 160 beats/minute) at different pulsed volumes (4 sets for 50 ml/beat, 3 sets for 70 ml/beat and 3 sets for 100 ml/beat).

Due to slight fluctuations in the phantom gel mechanical property over time, we observed small variations in measured strains (by both sonomicrometers and speckle tracking) between experiments using the same pulsed volume and pump rate, though this never exceeded 5 points of strain value. Radial strain values assessed by the two methods ranged approximately from 5% to 15% for a pulsed volume of 50 ml/beat, from 15% to 25% for 70 ml/beat and 25% to 35% for 100 ml/beat (Fig 4).

One-way analysis of variance documented a significant difference for the ratio RS-Spt/RS-SN across the 3 pulsed volumes tested in this study (P = 0.024). A post hoc analysis confirmed that this ratio obtained with 50 ml/beat was significantly higher from the two other ones obtained with 70 and 100 ml/beat. The two-way analysis of variance testing the interactions of deformation rate and strain magnitude did not result in any significant effect on deformation rate (P = 0.54) nor interaction between these two factors (P = 0.84). Finally, to ensure that the accuracy of the tracking observed in in the upper wall was not segment-dependent, radial and circumferential strain speckle values of the different walls were compared at 80, 120 and 160 beats/minute. The results are displayed in Table 2. The standard deviation between lateral segments was greater for radial strain than circumferential strain and vice-versa for the upper and lower segments, regardless the pump rate.

**Table 2:** Segmental circumferential and radial speckle tracking-derived strain in wall segments at different pump rates.

Pump rate (beats/min)	Circumferential strain (%)			Radial strain (%)		
	Average of 6 segments	Upper and lower segments	Lateral segments	Average of 6 segments	Upper and lower segments	Lateral segments
80	22.8 ± 2.1	22.5 ± 3.5	22.9 ± 1.8	30.0 ± 1.7	30.2 ± 1.9	29.8 ± 1.9
120	24.0 ± 1.0	24.5 ± 1.9	23.7 ± 0.5	32.3 ± 2.8	32.0 ± 1.9	32.5 ± 3.4
160	24.2 ± 2.0	23.9 ± 2.4	24.3 ± 2.2	31.8 ± 2.3	31.7 ± 1.5	31.9 ± 2.9

Values are expressed as mean ± SD.

## Discussion

The need for accurate assessment of myocardial deformation during stress echocardiography [4,25] has highlighted the importance of validating speckle tracking algorithms at high heart rates. Speckle tracking accuracy depends on image quality, and a frame rate ranging from 40 to 80 Hz is recommended to ensure a sufficient change in speckle patterns detectable by the algorithm [23]. Because of the tachycardia observed during a stress echocardiography test, there is a theoretical risk of undersampling and missing the peak of the maximal deformation. Moreover, although higher frame rates would reduce such risk, it would result in sub-optimal measures because speckle tracking methods perform better under large deformations as long as they remain within the kernel size of the block-matching algorithm [26]. Our in vitro study demonstrated, for a constant frame rate, a strong correlation between speckle tracking-strain and sonomicrometry-strain regardless of the pump rate used.

As expected, the accuracy of speckle tracking-strain was found to be weaker at low strain magnitudes with a minor but significant overestimation by the echocardiographic algorithm compared to sonomicrometry (a relative mean error of 3.3% for radial strain values <15%). This observation is similar to results obtained by Korinek J. et al. [12]. By looking at a longitudinal distortion of a tissue-mimicking gelatin block, the authors showed a tendency of speckle tracking to overestimate lower strain values compared to sonomicrometry. These results were explained in the latter study by subpixel displacements occurring at lower strain magnitudes.

In small animal studies [27,28] where the rapid heart rate and the small size of the heart require both high spatial and temporal resolutions, strain assessment by speckle tracking has been shown to be feasible using conventional echocardiography systems (GE, probe i13L-14Mhz), and helpful for detecting regional left ventricular dysfunction [28]. Reant et al. [29] have also demonstrated the potential feasibility and utility of speckle tracking-strain during stress conditions in a large animal model as well. In their study, the authors showed a good overall agreement between speckle tracking and sonomicrometry data at rest and under dobutamine infusion for the detection of inducible ischemia (frame rate 70–80 Hz). However, the accuracy of speckle tracking to assess the radial deformation in short axis view was found to be weaker and more variable compared with longitudinal and circumferential strains. During ischemia, radial function is altered later than longitudinal function but is known to be more difficult to assess in short axis view [30]. Langeland et al. [31] provided a possible explanation for this radial strain unreliability. By analyzing both the axial and azimuth radial strain components, they found that the correlation between speckle tracking and sonomicrometry were better for the axial-radial component compared to the azimuth one. This greater accuracy of the axial-radial strain is due to the intrinsic higher axial resolution of ultrasonic imaging. However, spatial resolution is lower in the transverse direction making perpendicular tracking less robust, which is the case for the assessment of radial strain within septal and lateral walls, in short axis view. Finally, our study showed a slightly higher variability of radial strain measurements in lateral segments compared to axial segments. However, these differences are too small to be of clinical consequence.

### Limitations

Our phantom model was not designed to reproduce the natural structural and functional modifications of natural fibers within an ischemic myocardium, since it was made of a homogenous mixture of graphite powder and polyvinyl alcohol. However this design still allowed us to technically verify whether or not major changes in hemodynamic conditions such the heart rate could dramatically affect the tracking of the algorhithm. The assessment of longitudinal strain rather than radial strain would have been a more realistic model in comparison with what is done during a stress echocardiogram. However, in addition to the technical complexity of building a phantom which could be scanned from the apex and which would able to tolerate high pump rates, we wish to highlight the point that the speckles in our phantom are linked only to graphite powder and not to myocardial fibers. Therefore, in addition to its homogeneous elasticity, our phantom has homogeneous acoustic properties, making the tracking of the upper wall in a short axis view technically equivalent to tracking longitudinally. Although sonomicrometry and echocardiography measured radial deformations of the same segment, speckle tracking assessed a mean radial deformation within a larger region of interest compared to the scanning line of aligned crystals. Thus, having a homogeneous vessel phantom likely helped to obtain high correlations between both methods. Reported results could also be over-optimistic because the contrast between the walls of the phantom and the surrounding fluid was marked and more important than in vivo scans. Consequently, it was of course easier for the algorithm to track the tissue in this phantom in comparison with patients. Finally, in this study, we tested only one commercially available strain analysis software.

### Clinical implications

Diagnosis of myocardial ischemia and coronary artery disease are of major concern for the management of patients in cardiology. The proposed in vitro validation of speckle tracking-strain at high heart rates could be translated into clinical practice since the quantitative assessment of myocardial deformation is known to be superior to a subjective evaluation of wall motion to detect inducible ischemia during stress echocardiography [32]. The present study confirms the clinical observations on the feasibility and accuracy of speckle tracking in assessing local deformation during stress echocardiography.

## Conclusions

In this in vitro study, we have demonstrated that a commercially available speckle tracking algorithm is accurate to assess axial deformations across a wide range of heart rates and strain magnitudes, as can be observed during stress echocardiography. This experimental study ensures the clinician that any change in strain during a stress echocardiography is more likely to be reflective of a true change rather than an artifact of the speckle tracking algorithm. Our results lay the foundation for future studies to determine the optimal integration of speckle tracking into clinical practice to improve the assessment of myocardial function during stress echocardiography and the diagnostic ability of the physician.

## Supporting information

S1 Video Video of the phantom model. For the purposes of optimizing video quality, the experimental setup was removed from the water.
(MOV)

S2 Video Example of the speckle tracking software assessing radial strain in short axis view in the phantom model. The post-processing analysis software was EchoPAC (BT12, GE Medical).
(AVI)
